# A new species of *Stamnaria* (Leotiomycetes, Helotiales) from Western Siberia

**DOI:** 10.3897/mycokeys.32.23277

**Published:** 2018-03-20

**Authors:** Danny Haelewaters, Nina V. Filippova, Hans-Otto Baral

**Affiliations:** 1 Organismic and Evolutionary Biology, Harvard University, 22 Divinity Avenue, Cambridge, Massachusetts 02138, USA; 2 Yugra State University, 628012, Chekhova Street, 16, Khanty-Mansiysk, Khanty-Mansiysk Autonomous Okrug, Russia; 3 Blaihofstraße 42, 72074 Tübingen, Germany

**Keywords:** Ascomycota, ecology, *Equisetum*, ITS rDNA, *Stamnaria*, taxonomy

## Abstract

A new species of *Stamnaria* is described based on morphology and molecular data from a collection made in West Siberia. *Stamnaria yugrana* is differentiated by lanceolate, strongly protruding paraphyses and comparatively narrow, fusoid-clavate ascospores. The apothecia are urn-shaped due to a prominent and even collar as in *S. persoonii*. The species grows on fallen side branches of *Equisetum sylvaticum*, a rarely recorded host for *Stamnaria*. The authors formally describe the new species and provide colour illustrations. In addition, the literature is reviewed on previously described species of *Stamnaria*. Phylogenetic reconstruction of the *Stamnaria* lineage, based on the ITS ribosomal DNA, strongly supports the three currently recognised species: *S. americana*, *S. persoonii* and *S. yugrana*.

## Introduction

During ongoing studies of the Helotiales (see Baral and Haelewaters 2015, Baral et al. 2015), material of a species of *Stamnaria* Fuckel, which had been collected and morphologically documented by one of the authors (NVF), was investigated by molecular methods. Collections were made during surveys in the vicinities of Shapsha field station of Yugra State University in Western Siberia, Russia. After the first collection of the species in June 2008, the same locality was visited in June 2012, February 2014 and May 2015. During all these visits, abundant apothecia were observed. The species was always found growing on *Equisetum sylvaticum*. This plant species had rarely been mentioned before as a host for the genus *Stamnaria* (Sydow 1898: 432, *S. equiseti*; Jaap 1922: 15, *S. persoonii*). Moreover, the microscopic features of the collections differed markedly from all earlier taxa described in the genus *Stamnaria*. Therefore, it was decided to formally describe this fungus as a new species based on morphological and molecular characteristics.

Members of the genus *Stamnaria* (Ascomycota, Leotiomycetes, Helotiales) share the following characteristics: 1) the presence of a thick hyaline gelatinised layer of textura oblita outside the ectal excipulum of textura prismatica, 2) cells of ectal excipulum and paraphyses containing yellow-orange carotenoids, 3) apothecia erumpent through epidermis, 4) all species growing on members of *Equisetum* and 5) with asexual state in the genus *Titaeospora* Bubák (von Höhnel 1928, Gruber 2006, Baral in Jaklitsch et al. 2016). According to Johnston et al. (2014), the older name *Stamnaria* is recommended to be used for the holomorph, instead of *Titaeospora*. Based on the currently available sequence data, the genus forms its own clade within the Helotiales (Baral and Haelewaters 2015). Its isolated position was emphasised by Baral in Jaklitsch et al. (2016) by the informal recognition as “*Stamnaria* lineage.”

Thus far, seven species have been referred to as *Stamnaria* in the literature: *S. americana* Massee & Morgan, *S. herjedalensis* (Rehm) Bubák, *S. hyalopus* P. Karst., *S. equiseti* (Hoffm.) Sacc., *S. persoonii* (Moug.) Fuckel, *S. pusio* (Berk. & M.A. Curtis) Massee and *S. thujae* Seaver, according to the Index Fungorum (2018). However, *S. hyalopus*, *S. pusio* and *S. thujae* do not belong in the genus *Stamnaria* because they grow on hosts other than *Equisetum*, amongst other reasons (Gruber 2006). *Stamnaria hyalopus* occurs on decaying leaves of *Carex vesicaria* and *S. thujae* on *Thuja occidentalis*. *Stamnaria pusio* grows on rotting debris in the soil and was placed in tentative synonymy with *Sarcoscypha occidentalis* (Schwein.) Sacc. by Harrington (1990). *Stamnaria herjedalensis* appears to be a synonym of *Roseodiscus equisetinus* (Velen.) Baral (O. Eriksson pers. comm.). *Stamnaria equiseti* is considered a synonym of *S. persoonii* by accepting the older name *equiseti* (Saccardo 1889) but *S. equiseti* is of uncertain identity whereas that of *S. persoonii* could be clarified based on the lectotype (Gruber 2006). Taken together, only two species are currently accepted in the genus *Stamnaria*: *S. americana* and *S. persoonii*.

## Material and methods

### Study site ecology

The study site is located in the middle taiga sub-zone of Western Siberia, in Russia. The area is characterised by a subarctic climate with average yearly temperatures of -1.1 °C, ranging from averages of -20 °C in January to 18 °C in July. The total annual precipitation is 553 mm. The period without snow cover usually lasts from May until October (Bulatov et al. 2007).

The collections were made in a mixed coniferous-deciduous forest close to a stream and a forest path 1.2 km SSE of Shapsha village, 61.07929"N, 69.46925"E. The tree canopy was dominated by *Pinus sibirica*, *Picea obovata* and *Abies sibirica* with admixture of *Betula pubescens*, *Populus tremula*, *Sorbus sibirica* and *Salix* spp. The plant layer was made up of *Equisetum sylvaticum*, *Rubus arcticus*, *Milium effusum* and *Luzula pilosa*.

Several years ago, a fire took place in this forest, resulting in the dominance of *E. sylvaticum*. Ground fires generally result in completely new successional trajectories and one dominant component of early post-fire vegetation communities is *E. sylvaticum*. Its rhizomes are buried deep in the soil and thus are resistant to fire (Viereck 1983). Interestingly, the new *Stamnaria* species was only collected from these post-fire mass populations of *E. sylvaticum*. Elsewhere in coniferous forests in Khanty-Mansi Autonomous Okrug – Yugra, *E. sylvaticum* is common but occurs in sparse densities and no growth of *Stamnaria* has been observed.

### Morphological studies

The species was discovered after examining the litter of the host plant *in situ* by the naked eye. Time of collection was always at the beginning of summer (about three weeks after snow melt). The substrate (*Equisetum sylvaticum* side branches) extracted from under the snow in February 2014 also gave abundant fruiting after three weeks of incubation in a moist chamber.

The litter was collected and brought to the laboratory where it was studied and documented the same day. Hereafter, the material (side branches with attached fruiting bodies) was dried at room temperature and stored as dry collection at Yugra State University Biological Collection (YSU). Voucher specimens are also preserved at Farlow Herbarium of Harvard University, Cambridge, Massachusetts (FH) and at V.L. Komarov Botanical Institute, Saint-Petersburg (LE).

The morphological features of the species were studied using a Zeiss Stemi 2000-C stereomicroscope, with magnification from 6 to 50× and a Zeiss Axiostar transmitted light microscope (with Achromat 10/0.25, 40/0.65 dry and 100/1.25 oil immersion objectives). Microstructures were studied and measured from living material in tap water and later compared to dead material from dried specimens. The iodine reaction was tested with Lugol’s solution and Congo Red in water was used to stain the sections and the structure of the excipulum.

Macro- and micro-photographs were obtained using a Canon EOS 50D digital camera and Axiocam ERc 5s digital camera. Abbreviations used: * = living state, † = dead state, CR = Congo Red, VB = vacuolar body.

### DNA extraction, PCR and sequencing

DNA was extracted from dry apothecia with the help of the Extract-N-Amp Plant PCR Kit (Sigma-Aldrich, St Louis, MO), DNeasy Plant Mini Kit (Qiagen, Valencia, CA) and the QIAamp DNA Micro Kit (Qiagen). Per extraction, 1 to 4 apothecia were used. Apothecia were crushed in 1.5 mL tubes using a 1.5 mL pellet pestle (Kimble, Rockwood, TN, #749521-1500) or cut in half using a sterile no. 10 surgical blade on a disposable Bard-Parker handle (Aspen Surgical, Caledonia, MI). Undiluted DNA was used for PCR amplification of the internal transcribed spacer (ITS) region of the ribosomal DNA (rDNA). The ITS was amplified using the forward primer ITS1f (5’–CTTGGTCATTTAGAGGAAGTAA–3’) in combination with either ITS4 (5’–TCCTCCGCTTATTGATATGC–3’) or the Ascomycota-specific primer ITS4A (5’–CGCCGTTACTGGGGCAATCCCTG–3’) (White et al. 1990, Gardes and Bruns 1993, Larena et al. 1999). All PCR reactions were done in a Mastercycler ep gradient thermocycler (Eppendorf, Hauppauge, NY) using Sigma-Aldrich’s REDTaq DNA Polymerase enzyme. PCR conditions were as follows: an initial denaturation step at 94 °C for 3 min, then 35 cycles of 94 °C for 1 min, 50 °C for 45 s and 72 °C for 90 s and a final extension step at 72 °C for 10 min (ITS).

Products with clear bands on agarose gel were cleaned with the Qiaquick PCR Purification Kit (Qiagen) and subsequently sequenced with the same primers (3 μl of purified PCR product per 10 μl sequencing reaction). Sequencing reactions were performed using the Big Dye Terminator v3.1 Cycle Sequencing Kit (Life Technologies, Carlsbad, CA). Sequences were trimmed, edited and assembled in Sequencher v. 4.10.1. All generated sequences have been deposited in GenBank (Table 1). BLAST searches for similar sequences were undertaken at: http://ncbi.nlm.nih.gov/blast/Blast.cgi.

**Table 1.** Isolates used in phylogenetic analyses, with voucher information and GenBank accession numbers. Accession numbers of sequences generated during this study are in bold. *This sequence was retrieved from the Biological Resource Center of the National Institute of Technology and Evaluation, Japan (NBRC).

Species	Isolate	Voucher	GenBank accession number
*Geoglossum nigritum*	AFTOL-ID 56	OSC 100009	DQ491490
*Geoglossum umbratile*	ANM Acc377	ILLS:61040	JQ256422
*Hymenoscyphus epiphyllus*	–	H.B. 7054	DQ431180
*Leotia lubrica*	ZW-Geo59-Clark	–	AY789360
*Microglossum rufum*	–	–	AY144533
*Rommelaarsia flavovirens*	E5	H.B. 9684	KT958772
*Roseodiscus rhodoleucus*	DH257	H.B. 8488a	KT972704
*Sarcoleotia globosa*	–	OSC 63633	AY789410
*Stamnaria americana*	DH258a	H.B. 7261	KT972707
	DH941c	Gruber 152/226	**MG662188**
	DH941d	Gruber 152/226	**MG662189**
	FC-2732	TNS-F-39244	NBRC 108774*
*Stamnaria persoonii*	DH671a	Gruber 119/183	**MG662201**
	NLU003b	Gruber 118/182	**MG662202**
*Stamnaria yugrana* sp. nov.	DH603a	FH 01146308	**MG662203**
	DH603b	FH 01146308	**MG662204**

### Sequence alignment, nucleotide divergence and phylogenetic analyses

An ITS dataset was constructed to investigate the phylogenetic structure within the genus *Stamnaria*. Sequences were aligned using Muscle v3.7 (Edgar 2004), available on the Cipres Science Gateway version 3.3 (Miller et al. 2010). Pairwise evolutionary distances, the amounts of genetic variation between two species, were calculated using Paup on XSEDE, also on the Cipres Science Gateway. The p-distance is calculated as the proportion of sites in a pairwise alignment at which the compared sequences are different. This is the number of nucleotide differences divided by the number of nucleotides compared. In addition, Jukes-Cantor (JC69) and Kimura 2-parameter (K2P) distance metrics (Jukes and Cantor 1969, Kimura 1980) were calculated. Neighbour joining (NJ) analyses (Saitou and Nei 1987) were performed to cluster taxa based on the respective distance matrices. The three resulting NJ trees were statistically compared with the SH test (Shimodaira and Hasegawa 1999).

Maximum parsimony (MP) and maximum likelihood (ML) analyses were run using Paup on XSEDE. MP was estimated with heuristic searches consisting of 500 stepwise-addition trees obtained using random sequence addition replicates followed by tree bisection-reconnection (TBR) branch swapping (MulTrees in effect) and saving all equally most parsimonious trees. Robustness of branches was estimated by maximum parsimony bootstrap proportions (BP) using 1000 bootstrap replicates, with heuristic searches consisting of 10 stepwise-addition trees obtained using random sequence addition replicates followed by TBR branch swapping, with MaxTrees set at 100. ML inference was run under a TIM2ef+I+G model of nucleotide substitution, as selected by jModeltest 2.1 (Darriba et al. 2012) following the Akaike Information Criterion. Rapid bootstrapping was implemented with 500 replicates.

Bayesian analyses were done with a Markov chain Monte Carlo (MCMC) coalescent approach implemented in Beast 1.8.4 (Drummond et al. 2012), with a strict clock with uniform rates of evolution across branches. A Yule speciation (Yule 1925) tree prior was selected with the TIM2ef+G nucleotide substitution model (considering the Bayesian Information Criterion from the earlier jModelTest 2.1 analysis). Four independent runs were performed from a random starting tree for 40 million generations, with a sampling frequency of 4000. Tracer 1.6 (Rambaut et al. 2014) was used to check trace plots and effective sample sizes (ESS). Burnin values were 5 % for all runs. Logged parameters were checked to have combined ESS values of at least 200. TreeAnnotator 1.8.4 was used to generate consensus trees with 0 % burnin and to infer the Maximum Clade Credibility (MCC) tree, with the highest product of individual clade posterior probabilities. The different trees with bootstrap values (BS) and posterior probabilities (pp) were visualised in FigTree 1.4.3 (http://tree.bio.ed.ac.uk/software/figtree/).

## Results

### Taxonomy

#### Stamnaria yugrana

Taxon classificationFungiHelotialesHelotiaceae

Filippova, Haelew. & Baral sp. nov.

823742

##### Diagnosis.

Characterised by the presence of both lanceolate and cylindrical paraphyses, fusoid-clavate ascospores with a length/width ratio of predominantly >4 and free-ending hyphae at the inner excipulum of the tube-shaped, even collar. Saprophytic on dead branches of *E. sylvaticum*.

**Figure 1.**
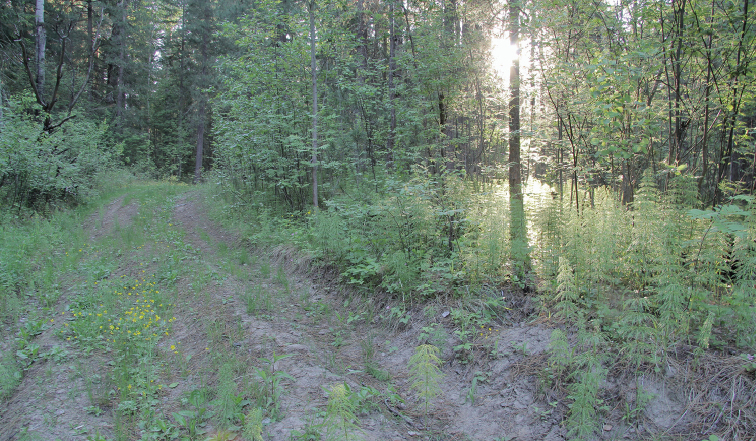
Study site of *Stamnaria yugrana* growing on litter of *Equisetum sylvaticum* in Western Siberia near the Khanty-Mansiysk town.

##### Types.

Holotype: Russia, Western Siberia, Khanty-Mansi Autonomous Okrug – Yugra, 25 km ENE of Khanty-Mansiysk town, 1.2 km SSE of Shapsha village, 61.07929"N, 69.46925"E, alt. 40 m, 9 Jun 2012, *leg.* N.V. Filippova, on fallen side branches of *Equisetum sylvaticum* L. lying amongst other forest litter in a mixed coniferous-deciduous forest; Biological Collection of Yugra State University (YSU-F-03519). Isotypes: LE-295215; FH 01146308. Paratypes: *ibid.*, 16 Jun 2008 (YSU-F-00097, material lost; LE-295060); *ibid.*, 22 Feb 2014, substrate collected from under snow and grown in a moist chamber (YSU-F-04933); *ibid.*, 25 Feb 2015 (YSU-F-06579; LE-296061).

**Figure 2.**
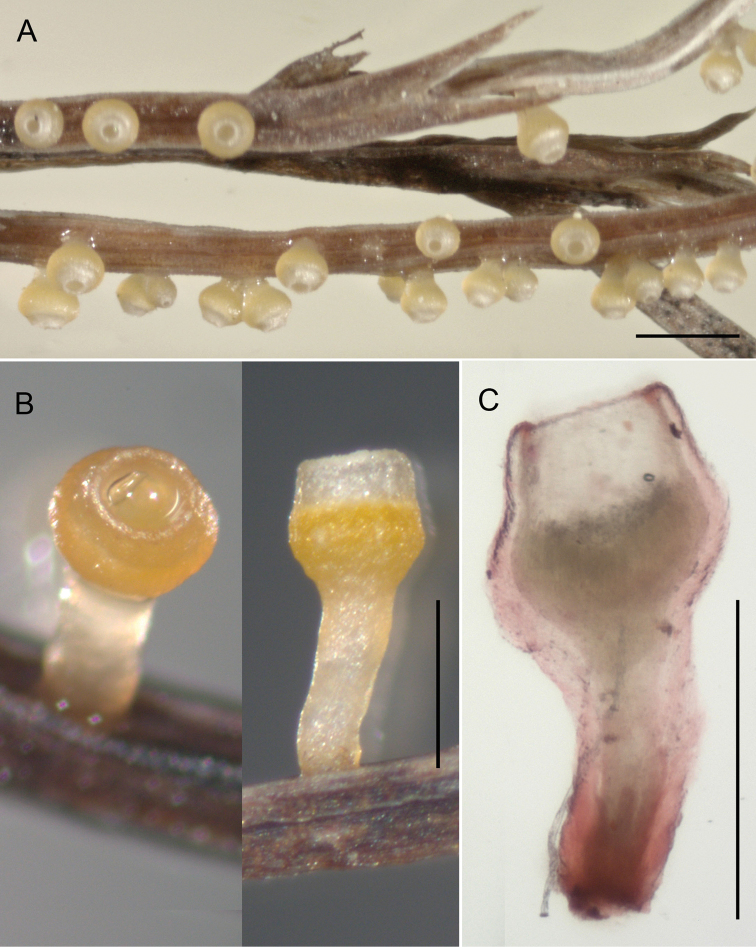
Apothecia of *Stamnaria yugrana* on side branches of *Equisetum sylvaticum*: **A** Apothecia grown *in situ* under well-lit conditions **B** Apothecia grown in shady conditions after incubation in a moist chamber **C** Median section through an apothecium after incubation in a moist chamber (dead, in CR). A from YSU-F-03519, B from YSU-F-04933, C from YSU-F00097. Scale bars: **A** 1.0 mm, **B, C** 0.5 mm.

##### Description.

***Apothecia*** urn-shaped, stipitate, 0.25–0.6 mm in diameter when mature, 0.5–1 mm high, varying depending on light conditions, being stouter with shorter stipe when substrate exposed to sunlight; receptacle light yellow(-ochraceous) when fresh, with even, whitish collar ~80–120 µm high, stipe pale yellowish-translucent, 100–380 × 130–300 µm, receptacle becoming light brown on drying; scattered to moderately gregarious, often abundant on the branches. ***Ectal excipulum*** outer layer *40–45 µm thick at middle flanks and margin, made up of strongly gelatinous tissue of loose, parallel to wavy hyphae 2–3 μm broad, septate, embedded in abundant gelatinous matrix (textura oblita); inner layer *~45 µm thick at middle flanks, made up of textura prismatica-porrecta running parallel to outside, cells at middle flanks *17–44 × 7–12 μm, slightly narrower in the collar region, much narrower in stipe (*20–45 × 3–5 µm); the inner layer of the collar composed of narrow hyphae, free upper part of these hyphae internally covered by lateral cellular outgrowths *2–5 × 1.5–2 µm. ***Medullary excipulum*** well developed, of dense, parallel, septate hyphae (textura porrecta) without gel, cells *65–90 × 2.5–6 (–7.7) μm; subhymenium well developed (*20–30 thick), of intricate hyphae *2 μm broad. ***Asci*** cylindrical, developing from croziers which are difficult to see in mature asci, with apical thickening enclosing a hemiamyloid ring of *Calycina*-type (rb: dirty red at high, blue at low concentration), *146 × 12.5 [123–159 (–206) × 11.7–13.5] μm, †98 × 9 (90–110 × 8–10.5) μm, 8-spored, spores *obliquely biseriate. ***Paraphyses*** of two types: (1) lanceolate, exceeding asci for *12–20 µm when young and *30–40 μm when fully developed, septate in lower part, non-septate in broad upper part, with quite acute tip, in young paraphyses with granular (multiguttulate) vacuolar content of moderate refractivity (VBs), later replaced by larger non-refractive vacuoles, *5–7 (†3–6) μm broad in upper part; (2) cylindrical, more abundant, not exceeding the asci, *2.3–3 μm broad above, septate, with obtuse tip, rarely branched below and scarcely enlarged in upper segment, without VBs, with pale yellow-orange pigment in middle and lower part. ***Ascospores*** fusoid-clavate, slightly to distinctly heteropolar, with rounded to obtuse ends, usually without any gel around, filled with granular oil content in both halves, leaving a central zone for the single nucleus, variable in length, *19.8 × 4.8 (16.5–24.5 × 4.2–5.6), n=18, Q=4.1 (YSU-F-04933); †20.5 × 4.0 (17.2–24.2 × 3.6–4.6) μm, n=37, Q=5.1 (YSU-F-03519, YSU-F-04933).

**Figure 3.**
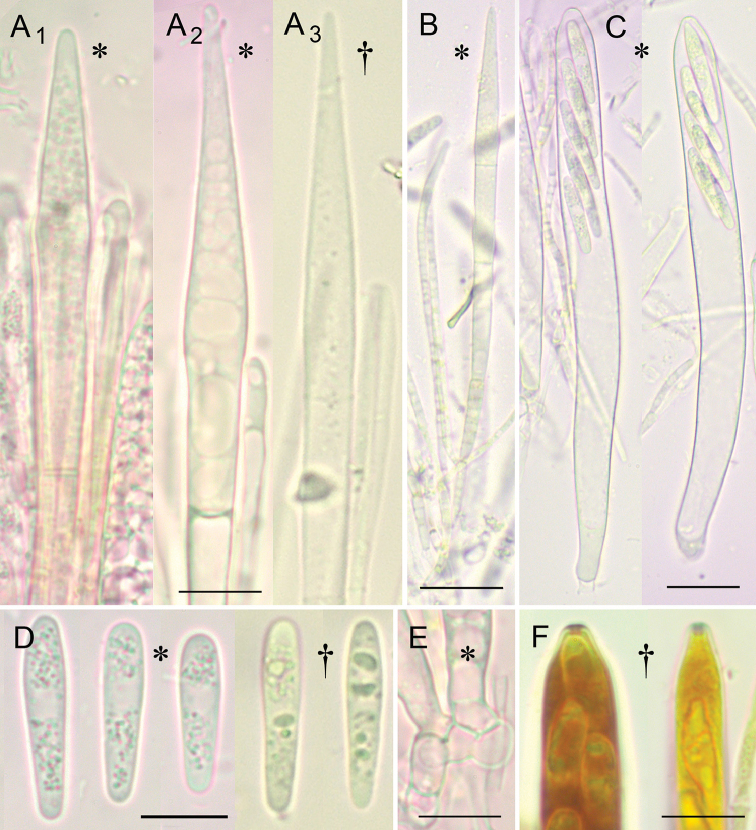
**A_1_** Young living paraphysis with multiguttulate, medium refractive vacuolar content (VBs), **A_2_** Mature living paraphysis with large non-refractive vacuoles **A_3_** Dead paraphysis with small oil drops (LBs), vacuoles disappeared **B** Paraphyses of cylindrical and lanceolate type **C** Mature living asci **D** Mature ejected ascospores with granular lipid content of minute LBs (confluent in dead state) **E** Ascus bases with croziers **F** Apices of mature and immature ascus (stained in IKI). All * and F from YSU-F-04933, all other † from YSU-F-00097. Scale bars: **A, D, E, F** 10 μm, **B, C** 20 μm.

##### Etymology.

Referring to Yugra, the historical name of the region (currently “Khanty-Mansi Autonomous Okrug – Yugra”).

##### Distribution.

Known only from the type locality.

### Nucleotide alignment dataset and phylogenetic inferences

The ITS rDNA dataset consists of 16 isolates and 671 characters, of which 387 are constant and 187 are parsimony-informative. Taxonomical sampling covers the genera *Hymenoscyphus* Gray (1 isolate), *Leotia* Pers. (1), *Microglossum* Gillet (1), *Rommelaarsia* Baral & Haelew. (1), *Roseodiscus* Baral (1) and *Stamnaria* (8); *Geoglossum* Pers. (2) and *Sarcoleotia* S. Ito & S. Imai (1) served as outgroup taxa (Geoglossomycetes, Geoglossales, *sensu* Schoch et al. 2006). This dataset includes the three currently recognised species in the genus *Stamnaria*.

**Figure 4.**
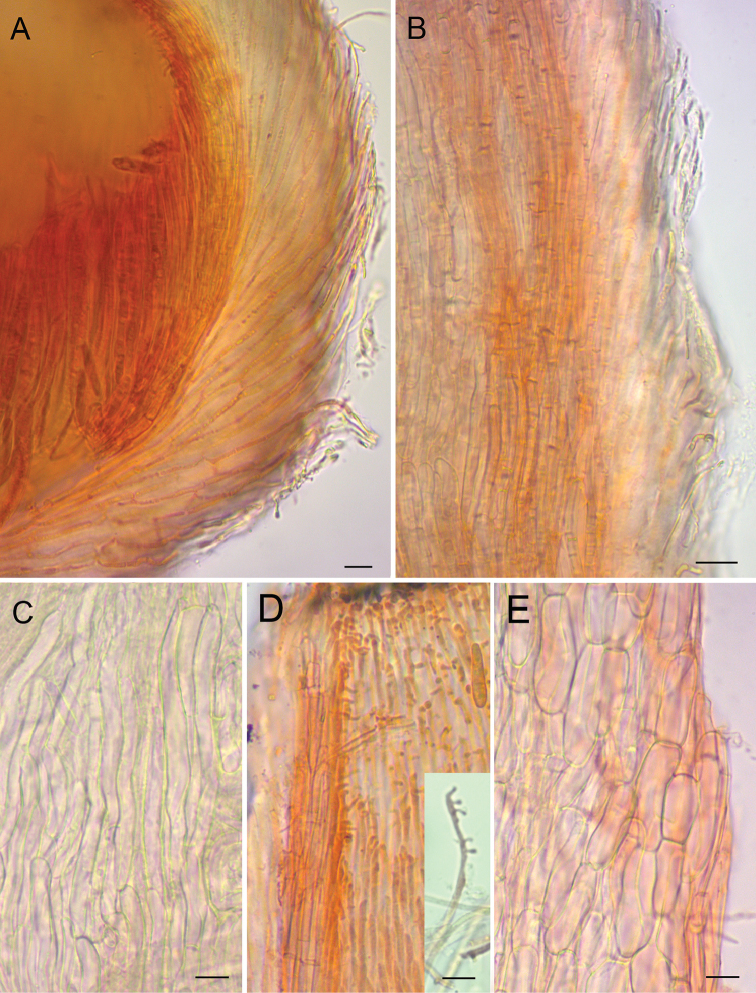
**A** Median section through receptacle, showing gelatinised outer layer of ectal excipulum **B** Median section through stipe, showing outer gelatinised layer, inner layer of textura porrecta (orange) and transition to medullary excipulum **C** Medullary excipulum in squash mount **D** Inner part of collar in squash mount showing hyphae forming outgrowths (single hyphae shown in insert) **E** Ectal excipulum of textura prismatica at receptacle flank in squash mount (outer layer absent). All stained by CR in water (cells partly in vital state), from YSU-F-04933. Scale bars: 10 μm.

Intra-speciﬁc divergence in the ITS region ranges from 0.0 to 0.9 % while inter-speciﬁc divergences range between 8.0 and 13.7 % (p-distances) or between 8.4–8.5 and 15.1 % (JC69, K2P). The p-distances are 8.0 % between *S. americana* and *S. persoonii*, 10.6 % between *S. persoonii* and *S. yugrana* and 13.7 % between *S. americana* and *S. yugrana*. JC69 and K2P distances are almost identical, higher but equivalent to the p-distances (Table 2). The NJ phenogram constructed from the Jukes–Cantor distance matrix has a higher likelihood score (-lnL = 791.63111) compared to the analyses based on p-distances and Kimura 2-parameter distances, but the differences are insignificant.

**Table 2.** Intra- and interspecific distances for and between *S. americana*, *S. persoonii* and *S. yugrana*. Distances are given as percentages (%).

	**p-distance**	**JC69**	**K2P**
*S. americana*, intraspecific	0.2	0.2	0.2
*S. persoonii*, intraspecific	0.9	0.9	0.9
*S. yugrana*, intraspecific	0.0	0.0	0.0
*S. americana*–*S. persoonii*	8.0	8.4	8.5
*S. americana*–*S. yugrana*	13.7	15.1	15.1
*S. persoonii*–*S. yugrana*	10.6	11.4	11.4

The genus *Stamnaria* is retrieved as a monophyletic clade in all three phylogenetic reconstructions (MP BS = 74, ML BS = 98, pp = 1.0). All morphologically delineated species of *Stamnaria* have maximum support. The position of the new species within the genus is unresolved. Under MP and ML inference, *S. yugrana* is sister to *S. persoonii* but this sister relationship is only supported by ML BS = 81. In the Bayesian analysis, on the other hand, *S. yugrana* is sister to (*S. americana*, *S. persoonii*), with moderate support for the sister relationship between the two latter species (pp = 0.8).

**Figure 5.**
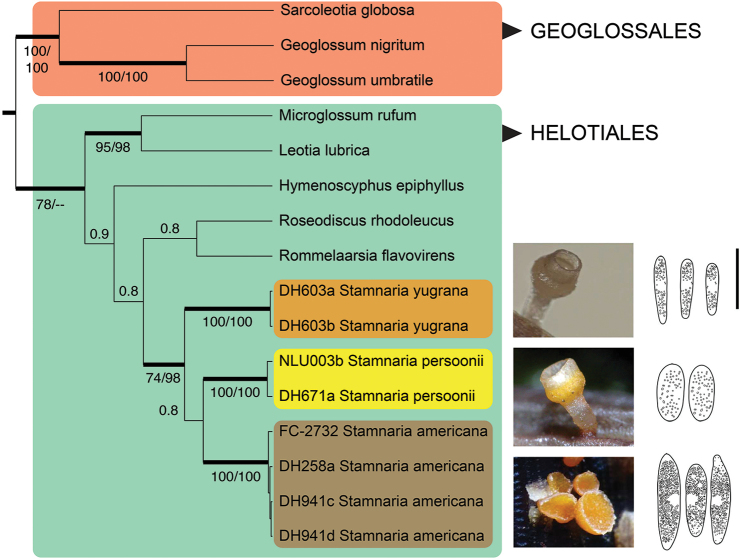
Bayesian MCC tree, with node values indicating posterior probabilities (above) and MP/ML bootstrap values (below). Thick branches indicate maximum Bayesian support (pp = 1.0). Photos of apothecia (left) and drawings of ascospores (right) from top to bottom: *S. yugrana*, holotype, YSU-F-03519, Russia, 9 Jun 2012; *S. persoonii*, Gilbert Moyne, H.B. 8889, France, 24.VI.2008; *S. americana*, Claude Page, France, 7 Apr 2016 (first French report in Moingeon and Page 2003). Scale bar: 20 µm.

## Discussion

The morphological and ecological features of *S. yugrana* (Table 3) are considerably different compared to the two previously recognised species, the most pronounced features being paraphyses of two types, including strongly protruding, broadly lanceolate ones and heteropolar, comparatively narrow ascospores. No previous reports of a *Stamnaria* species with ascertained identity are known from *E. sylvaticum*, when applying the revised species concept as reviewed in the introduction. Jaap (1922) presented a report of *S. persoonii* on *E. sylvaticum* but did not provide a morphological description. It is impossible to assess whether this report represents *S. persoonii* or *S. yugrana* because the collection does not seem to have been preserved (no voucher number cited).

*Stamnaria yugrana* differs from *S. americana* by solitary, never fasciculate, distinctly stalked apothecia, presence of a pronounced raised collar with free-ending hyphae at its inner excipulum, presence of two types of paraphyses (cylindrical and lanceolate) and shorter and especially narrower, fusoid-clavate ascospores (Table 3). The ecology of these two species is also quite different: the apothecia of *S. americana* occur on living stems of *Equisetum hyemale*, while those of *S. yugrana* are found on dead fallen side branches of *E. sylvaticum*. Both *S. yugrana* and *S. persoonii* have stipitate apothecia with a distinct collar. However, *S. yugrana* differs by the presence of paraphyses of two types and much narrower, heteropolar spores with higher length/width ratio (Table 3). Apothecia of *S. persoonii* also grow on dead stems, but on another host plant, *E. fluviatile* (Gruber 2006).

**Table 3.** Comparison of the ecological and morphological characteristics between described *Stamnaria* species.

Species	*S. americana*	*S. persoonii*	*S. yugrana*
**Host association**	Parasitic on *E. hyemale*	Saprophytic on *E. fluviatile*, rarely on *E. arvense*	Saprophytic on *E. sylvaticum*
**Apothecia, diameter**	0.3–0.7 (–1.0) mm	0.4–1.0 mm	0.25–0.6 mm
**Apothecia, margin**	Without collar	With even collar	With even collar
**Asci, measurements**	*(110–) 140–190 (–210) × 13.5–14.6 μm, †120–157 × (11.5–) 12–14 (–15) μm	*190–230 × 14–16 μm, †130 × 12 μm	*123–159 (–206) × 11.7–13.5 μm, †90–110 × 8.0–10.5 μm
**Asci, apex**	Inamyloid, thin-walled	With thick amyloid ring	With thick amyloid ring
**Ascospores, measurements**	*(22–) 24–28 (–34) × (6–) 6.5–7.5 (–8.4) μm, †20–29 × 5.5–7.0 μm	*16–23 × 7.5–9.5 μm, †15–18 × 5.0–8.0 μm	*16.5–24.5 × 4.2–5.6 μm, †16.5–23.2 × 4.0–4.8 μm
**Ascospores, shape**	Fusoid, not or only slightly heteropolar	Broadly ellipsoid with rounded ends	Fusoid-clavate
**Paraphyses**	Cylindrical, with apical part enlarged to *4.0–6.7 μm	Cylindrical, with apical part enlarged to *2.5–4.5 µm	Lanceolate, strongly exceeding, *5–7 (†3–6) μm broad; and cylindrical, not exceeding, *2.3–3.0 μm broad above
**Reference**	Künkele et al. (2005)	Dennis (1968), H.-O. Baral (pers. obs.)	This paper

Many *Stamnaria* collections have been misidentified and/or reported under misidentified host plants. Currently, the *Stamnaria* lineage has no taxonomic assignment at the family level. In their ITS+LSU rDNA phylogeny, Baral et al. (2015) retrieved it as a sister clade to Erysiphales, but without support for this sister relationship. Baral and Haelewaters (2015) found a sister relationship of *Stamnaria* and *Roseodiscus*, although only moderately supported by Bayesian analysis (pp = 0.83). Hosoya et al. (2013) suggested a transfer of the genus *Stamnaria* from Helotiaceae to the small family Leotiaceae. This was based on the strongly supported sister relationship with *Leotia lubrica* (Scop.) Pers. obtained in the maximum parsimony multigene analysis using rDNA, *EF1* and *RPB* genes, with bootstrap values of 100 from neighbour-joining and 99 from maximum parsimony. Morphologically, Hosoya et al. (2013) mentioned “a gelatinized layer in the external part of apothecia” as a synapomorphic character and suggested to include *Stamnaria* in Leotiaceae in a restricted sense, which traces back to a concept introduced by Korf & Lizoň (2001). However, recent molecular research indicated that the family Leotiaceae includes solely soil-inhabiting species with large ascomata with convex, pileate or clavate fertile part. Amongst the genera in this family, *Leotia* Pers. is the only one showing a gelatinised external layer outside the ectal excipulum (Baral in Jaklitsch et al. 2016). In addition, the ascospores of Leotiaceae consistently contain large oil drops (Baral in Jaklitsch et al. 2016), unlike *Stamnaria*.

Detailed morphological studies in the genus *Stamnaria* by Gruber (2006) revealed several other species, including collections that had been misidentified as *S. americana* or *S. persoonii* (*sensu* Magnes and Hafellner 1991, Brunelli 1992, Künkele et al. 2005). The author proposed several new names for species that he considered undescribed, but they have not been validly published. Formal descriptions of these new species along with molecular phylogenetic analyses are planned in the near future. It is clear that the genus *Stamnaria* is in need of thorough revision. The discovery and detailed description of *S. yugrana* will help in the further delimitation of the genus.

## Supplementary Material

XML Treatment for Stamnaria yugrana
